# Presence of *Legionella* and Free-Living Amoebae in Composts and Bioaerosols from Composting Facilities

**DOI:** 10.1371/journal.pone.0068244

**Published:** 2013-07-02

**Authors:** Lisa Conza, Simona Casati Pagani, Valeria Gaia

**Affiliations:** Swiss National Reference Centre for *Legionella*, Cantonal Institute of Microbiology, Bellinzona, Switzerland; University of Louisville, United States of America

## Abstract

Several species of *Legionella* cause Legionnaires’ disease (LD). Infection may occur through inhalation of *Legionella* or amoebal vesicles. The reservoirs of *Legionella* are water, soil, potting soil and compost. Some species of free-living amoebae (FLA) that are naturally present in water and soil were described as hosts for *Legionella*. This study aimed to understand whether or not the composting facilities could be sources of community-acquired *Legionella* infections after development of bioaerosols containing *Legionella* or FLA. We looked for the presence of *Legionella* (by co-culture) and FLA (by culture) in composts and bioaerosols collected at four composting facilities located in southern Switzerland. We investigated the association between the presence of *Legionella* and compost and air parameters and presence of FLA. *Legionella* spp. (including *L. pneumophila*) were detected in 69.3% (61/88) of the composts and FLA (mainly *Acanthamoeba*, *Vermamoeba*, *Naegleria* and *Stenamoeba*) in 92.0% (81/88). *L. pneumophila* and *L. bozemanii* were most frequently isolated. FLA as potential host for *Legionella* spp. were isolated from 40.9% (36/88) of the composts in all facilities. In *Legionella*-positive samples the temperature of compost was significantly lower (*P* = 0.012) than in *Legionella*-negative samples. Of 47 bioaerosol samples, 19.1% (9/47) were positive for FLA and 10.6% (5/47) for *L. pneumophila*. Composts (62.8%) were positive for *Legionella* and FLA contemporaneously, but both microorganisms were never detected simultaneously in bioaerosols. Compost can release bioaerosol containing FLA or *Legionella* and could represent a source of infection of community-acquired *Legionella* infections for workers and nearby residents.

## Introduction

Legionnaires’ disease (LD) and Pontiac fever may occur after inhalation of bioaerosols contaminated with *Legionella*. The environmental reservoirs of *Legionella* are ground water, lakes and rivers [1], [2]; *Legionella*, however, was also isolated from potting soils and composts [3], [4], [5]. Most LD infections are linked to contaminated hot water systems, cooling towers [6], and air-conditioning systems [7], but LD cases related to potting soil use or gardening have also been described [4].

Free-living amoebae (FLA) are present in water and soil worldwide with at least two developmental stages [8]. Trophozoites feed on bacterial cells and reproduce; cyst are resistant amoebal forms able to survive in stress conditions [8]. Some FLA have been described as potential hosts for *Legionella*: species of *Acanthamoeba*, *Naegleria* and *Vermamoeba* (synonym of *Hartmannella*) support the intracellular growth of *Legionella* [9]; some species in these genera are opportunistic human pathogens [10].

FLA like *Acanthamoeba* are isolated and cultivated on agar inoculated with bacteria such as *Escherichia coli* or *Klebsiella aerogenes* [10]. Amoebal enrichment can be used to isolate and co-culture *Legionella* from natural freshwaters and clinical specimens [11], [12] and to improve the sensitivity of culturing [13]. *Legionella* and FLA may be present simultaneously in aquatic environments, hot water systems and cooling towers [14]: thus, FLA may play a role in amplification and protection of *Legionella* and could act as a vector in the transmission of LD.

Composting is widespread in industrialised countries. Facilities with structured organic waste management collect and store green waste in piles of ground material. The piles are mixed regularly and the composting process lasts approximately 1–2 years [15], during which the organic matter is biodegraded to produce material that is further reused as soil fertiliser in agriculture or gardening activities [16]. Compost, on the other hand, may develop unpleasant odours and dust and it can be potentially harmful to human health through the dispersion of bioaerosols produced by handling of organic waste [17], [18].

Bioaerosols contain particulate organic matter, water droplets and live or dead microorganisms, such as spores, fungi, bacteria or protozoa, allergens or toxins [18]. The small size of the particulate material allows bypassing the nasal mucosa and penetrating the lungs [18]. Bioaerosol emission rates and dispersal at composting sites are influenced by many factors, including compost temperature, sorting, shredding and turning of the piles [16], [19], geographic area, topography, meteorological conditions (e.g. temperature, humidity, wind and weather), and the composition of the source organic material [16], [17].

Composting facilities and their associated bioaerosols are therefore an interesting model to better understand the spread of *Legionella* and its vectors from compost piles to bioaerosol. The aim of this study is to investigate whether or not the compost may be a reservoir of *Legionella* and FLA and the composting facilities could be considered sources of transmission of community-acquired LD and Pontiac Fever through development of bioaerosols containing *Legionella* or FLA. In addition, we have investigated the correlation between the presence of *Legionella* and the presence of amoebae and selected environmental factors.

## Materials and Methods

We investigated 4 composting facilities (CF) in southern Switzerland (Canton Ticino); these are characterised by a structured, standardised handling of the green material and are distributed over the territory. We thank the managers of the 4 composting facilities for allowing sampling in their centres for use in our study.

A total of 88 samples were collected between March and November 2009. The facilities 1, 3 and 4 (CF1, 3, 4) were monitored twice monthly, but due to logistic problems were visited only once in June and November and the sampling was not performed in October. In winter the facilities were not monitored: we concentrate sampling in the period when LD cases are more frequent. In all locations, air temperature and relative humidity (measured with an Omniport 20 device, E+E Elektronik, Austria), as well as compost temperatures at 30 cm in depth and pH were recorded (MP220 pH-meter, Mettler Toledo, Switzerland). Air samples (1 m^3^) were collected in 10 ml Page’s saline solution (PAGE) with a portable cyclonic air sampler (Coriolis μ, Bertin technologies, France) with a flow rate of 250 l/min for 4 min and stored at 4°C. At each sampling day, 4 bioaerosol samples were collected in each facility at 5 m distance from different piles (green waste, ground material, middle stage compost and finished compost). At CF2 only 1 compost and 2 bioaerosol samples could be collected because after June the mature compost pile had already been distributed for further use.

Two compost samples of 1 kg each were collected from each facility per each sampling date and stored in plastic bags at 4°C. Samples were taken from a depth of about 30 cm in the compost piles. A 5 g compost portion was suspended in 10 ml PAGE according to the method described by Casati et al. [5]. Then, 40 µl of the compost supernatant or 50 µl of the bioaerosol suspension were inoculated onto 1.5% non-nutrient agar (NNA) plates seeded with a layer of *E. coli* (ATCC 25922), incubated at 28°C and checked every day for amoebal growth [12]. NNA plates were incubated at 28°C, because in a preliminary test performed with potting soil stored at 4°C more FLA specie were recovered with incubation of the samples at 28°C compared to 35°C (data not shown). To identify the detection limits of NNA culture, 9 aliquots of 5 g compost, sterilised for 15 min at 121°C to eliminate all FLA potentially present, and 9 ml of sterile PAGE (to mimic air samples) were spiked with 1 ml of an *A. polyphaga* (strain ATCC 50362) suspension at dilutions of 1–1×10^7^ cells per 5 g of compost or per 10 ml PAGE. Sterile PAGE or compost were used as negative controls. After spiking, compost and PAGE were thoroughly mixed to distribute amoebae homogeneously in the samples and 9 ml of sterile PAGE were added to the compost. The compost suspensions were mixed during 30 min at room temperature and subsequently plated onto NNA.

After culture, trophozoites were gently scraped from the NNA plates and suspended in 3 ml PAGE. Genomic DNA was extracted using the DNeasy kit (Qiagen, Switzerland) following the manufacturer’s instructions. FLA were identified by PCR amplification and sequencing of 18S rRNA with an ABI prism 310 Genetic Analyzer (Applied Biosystems, Foster City, USA), by using the primer Ami6F1 and Ami9R described by Thomas et al. [12]. After amplification and sequencing of the obtained amplicons, sequences were compared to those present in available databases using BLAST.

Co-culture was carried out for the isolation of *Legionella* by using a PAGE suspension of axenic *A. polyphaga* [20]. A suspension of 900 µl of FLA (approx.10^5^ FLA/ml) was added to each well of a 24-well microplate (TPP, Techno Plastic Products AG, Trasadingen, Switzerland) and incubated for 1 h at 36°C. 100 µl of compost supernatant diluted 1 : 10^5^ in PAGE and bioaerosols (diluted 1 : 1000) were then added to each well. One well of each plate contained only *A. polyphaga* as negative control. After inoculation, the microplates were centrifuged at 1,000 *g* for 30 min and incubated during 7 days at 36°C in a moist chamber [21]. After 7 days the wells were scraped to detach the amoebal monolayer from the well bottom, and 20 µl samples were diluted 1 : 10 with 0.2 M HCl–KCl acid buffer (pH 2.2) and vortexed three times during 10 min at room temperature. After acid shock, 100 µl amount of each acid-treated sample was then plated on solid GVPC agar and incubated at 36°C for 5 days.

All potential *Legionella* colonies isolated from GVPC agar were identified by MALDI-TOF MS [22] and by slide agglutination (Legionella Slidex, bioMérieux, Switzerland). Serotyping of *Legionella pneumophila* (1 and non-1) isolates was performed by indirect immunofluorescence assay, using the monoclonal antibodies from the Dresden panel [23], [24].

Enrichment by co-culture does not allow a quantitative assessment of *Legionella* and FLA in compost and bioaerosol samples. All bioaerosol samples were analysed separately but the results of samples collected at the same site on the same sampling day were pooled and average values used for further analysis, because the aim of our study was to assess the overall composition of the *Legionella* and FLA populations of the bioaerosols of each facility.

Results of continuous variables are presented as mean ± standard deviation, frequencies in percentages. Statistical analysis was carried out with SPSS version 17.0 (SPSS Inc., Chicago, Illinois, USA). A Chi-square with Yates’ correction (when applicable) was used to compare *Legionella* categorical variables. The Student’ *t* test was used to compare means of continuous variables.

The limit of detection for direct culturing and co-culture of the spiked composts and air samples was defined as the fifth percentile of all analysed positive and negative samples. Mean and standard deviations of plaque forming units (PFU) values obtained were determined in two parallel experiments for both compost and air samples. All measurements were carried out in duplicate.

## Results

*Legionella* and FLA were detected in 61 and 81 of 88 composts and 5 and 9 of 47 bioaerosol pools examined, respectively. *Legionella* was detected by co-culture in 52.9% to 83.3% of the composts (68.2±13.3%) and in 8.3% to 18.2% of the bioaerosol pools (10.8±8.4%) with the exception of the CF2 samples, from which *Legionella* could never be isolated during the sampling period (Table 1).

**Table 1:** Number and percentage (in parentheses) of samples containing free-living amoebae (FLA) and *Legionella* spp. in the composting facilities (CF) studied.

	CF1	CF2	CF3	CF4	Mean ± standard deviation
**Compost**					
**N**	**24**	**17**	**24**	**23**	
FLA	20 (83.3)	17 (100)	23 (95.8)	21 (91.3)	92.6±7.1
*Legionella* spp.	15 (62.5)	9 (52.9)	20 (83.3)	17 (73.9)	68.2±13.3
FLA+*Legionella* spp.	12 (50)	9 (52.9)	20 (83.3)	15 (65.2)	62.8±15.1
FLA+*Legionella* spp. not detected	1 (4.2)	0 (0)	1 (4.2)	0 (0)	2.1±2.5
**Bioaerosol**					
**N**	**12**	**12**	**12**	**11**	
FLA	4 (33.3)	2 (16.7)	1 (8.3)	2 (18.2)	19.1±10.4
*L. pneumophila*	2 (16.7)	0(0)	1 (8.3)	2 (18.2)	10.8±8.4
FLA+*Legionella* spp.	0 (0)	0 (0)	0 (0)	0 (0)	0±0
FLA+*Legionella* spp. not detected	6 (50)	10 (83.3)	10 (83.3)	7 (63.6)	70.1±16.3

FLA were detected at frequencies ranging from 83.3% to 100% in the compost heaps (92.6±7.1%) and from 8.3% to 33.3% in bioaerosol pools (19.1±10.4%, Table 1). The 17 composts collected in CF2 were all positive for FLA.

Most compost samples analysed in this study (62.8%) were positive simultaneously for both *Legionella* and FLA; *Legionella* alone was detected in only 5.7% and FLA alone in 28.4% of the samples (Table 1). During the sampling period, microorganisms belonging to these two groups were isolated from bioaerosol pools in CF1, CF3 and CF4, but *L. pneumophila* and FLA were never recovered simultaneously from the bioaerosol samples.

The *Legionella* species recovered during this study are listed in Table 2. *L. pneumophila* and *L. bozemanii* were isolated in all CF. *L. pneumophila* mainly belonging to serogroups (sg) 3, 4, 5, 10, 12 and 15 were isolated from all compost samples at high percentages (35.3% –79.2%). *L. pneumophila* sg 1 (subgroups Philadelphia, France/Allentown, Benidorm and OLDA) was isolated in all facilities but CF2 from 11.4% of the compost samples. *L. pneumophila* other than sg 1 was isolated from bioaerosol samples in CF1, CF3 and CF4, while *L. pneumophila* sg 1 (subgroup OLDA) was detected only in CF4. Several species were present in the same sample. *L. feeleii* was occasionally found in CF2 (5.9%) and CF4 (4.3%), *L. micdadei* (5.9%) only in CF2 and *L. longbeachae* (4.2%) only in CF3.

**Table 2:** Number and percentage (in parentheses) of compost and bioaerosol pools containing *Legionella* species analysed by co-culture in the four composting facilities (CF).

	CF1	CF2	CF3	CF4
**Compost**				
**N**	24	17	24	23
N positive	15 (62.5)	9 (52.9)	20 (83.3)	17 (73.9)
*L. pneumophila* sg 1	2 (8.3)	0 (0)	5 (20.8)	3 (13)
*L. pneumophila* other thansg 1	14 (58.3)	6 (35.3)	19 (79.2)	14 (60.9)
*L. bozemanii*	1 (4.2)	2 (11.8)	2 (8.3)	1 (4.3)
*L. feeleii*	0 (0)	1 (5.9)	0 (0)	1 (4.3)
*L. longbeachae*	0 (0)	0 (0)	1 (4.2)	0 (0)
*L. micdadei*	0 (0)	1 (5.9)	0 (0)	0 (0)
**Bioaerosol**				
**N**	12	12	12	11
N positive	2 (16.7)	0 (0)	1 (8.3)	2 (18.2)
*L. pneumophila* sg 1	0 (0)	0 (0)	0 (0)	1 (9.1)
*L. pneumophila* other than sg 1	2 (16.7)	0 (0)	1 (8.3)	1 (9.1)

*L. pneumophila* sg 1: *L. pneumophila* serogroup 1; *L. pneumophila* other than sg 1: *L. pneumophila* other than serogroup 1.

The analysis of spiked samples allowed setting the detection limit of culture at 2×10^2^ *A. polyphaga* in 1 g of compost or 10^3^ *A. polyphaga* in 1 m^3^ air, respectively.

We isolated FLA from compost and bioaerosol pools in all facilities (Table 3). Species of *Vermamoeba*, *Naegleria* and *Acanthamoeba*, all potential hosts for *Legionella*, were isolated from 40.9% of the composts. In composts, an environmental *Stenamoeba* sp. was frequently detected in CF1 (37.5%), CF2 (64.7%) and CF4 (56.5%) and some *Naegleria* were most common in CF3 (45.8%).

**Table 3:** Number and percentage (in parentheses) of composts and bioaerosol pools containing free-living amoebae (FLA) species recovered by culture in the four composting facilities (CF).

	CF1	CF2	CF3	CF4
**Compost**				
**N**	24	17	24	23
N positive	20 (83.3)	17 (100)	23 (95.8)	21 (91.3)
***Acanthamoeba castellanii*** 1,2	2 (8.3)	1 (5.9)	1 (4.2)	0 (0)
***Acanthamoeba hatchetti*** 1,2	1 (4.2)	1 (5.9)	0 (0)	0 (0)
***Acanthamoeba lenticulata*** 2	1 (4.2)	0 (0)	1 (4.2)	0 (0)
***Acanthamoeba polyphaga*** 1,2	1 (4.2)	1 (5.9)	1 (4.2)	0 (0)
*Acanthamoeba* spp.	2 (8.3)	0 (0)	5 (20.8)	3 (13)
Echinamoeba thermatum*	0 (0)	0 (0)	0 (0)	1 (4.3)
*Flamella balnearia*	0 (0)	0 (0)	0 (0)	1 (4.3)
***Vermamoeba vermiformis*** 1	1 (4.2)	2 (11.8)	2 (8.3)	3 (13)
***Vermamoeba*** ** spp.** 1	0 (0)	0 (0)	2 (8.3)	0 (0)
*Learamoeba waccamawenis*	1 (4.2)	0 (0)	0 (0)	1 (4.3)
***Naegleria australiensis*** 1,2	0 (0)	0 (0)	3 (12.5)	2 (8.7)
***Naegleria gruberi*** 1	0 (0)	0 (0)	3 (12.5)	0 (0)
*Naegleria* spp.	2 (8.3)	1 (5.9)	5 (20.8)	1 (4.3)
*Singhamoeba horticola*	0 (0)	1 (5.9)	0 (0)	0 (0)
Soil amoeba AND32	0 (0)	0 (0)	0 (0)	1 (4.3)
*Stenamoeba* sp.	9 (37.5)	11 (64.7)	4 (16.7)	13 (56.5)
*Tetramitus* sp.	0 (0)	0 (0)	1 (4.2)	0 (0)
***Vahlkampfia avara*** 1	3 (12.5)	0 (0)	1 (4.2)	0 (0)
***Vahlkampfia enterica*** 1	1 (4.2)	1 (5.9)	1 (4.2)	0 (0)
*Willaertia magna*	0 (0)	0 (0)	2 (8.3)	0 (0)
**Bioaerosol**				
**N**	12	12	12	11
N positive	4 (33.3)	2 (16.7)	1 (8.3)	2 (18.2)
***Acanthamoeba jacobsi*** 2	1 (8.3)	0 (0)	0 (0)	0 (0)
*Acanthamoeba* spp.	0 (0)	1 (8.3)	0 (0)	0 (0)
*Flamella balnearia*	1 (8.3)	0 (0)	0 (0)	0 (0)
***Vermamoeba vermiformis*** 1	2 (16.7)	0 (0)	0 (0)	0 (0)
*Naegleria americana*	0 (0)	1 (8.3)	0 (0)	0 (0)
*Naegleria* spp.	1 (8.3)	1 (8.3)	0 (0)	0 (0)
*Platyamoeba placida*	0 (0)	0 (0)	0 (0)	1 (9.1)
*Stenamoeba* sp.	1 (8.3)	0 (0)	1 (8.3)	0 (0)
*Tetramitus* sp.	0 (0)	0 (0)	0 (0)	1 (9.1)

0: FLA not detected;

1 FLA supporting intracellular growth of *Legionella* [9], [33];

2 opportunistic pathogenic FLA [33]; * *Echinamoeba thermatum* has a nucleotide identity of 75% with a corresponding database entry, which presumably is even not sufficient for a reliable identification at the genus level.

The pH of compost heaps varied between pH 6.3 and 8.7 (7.6±0.44) and the temperatures between 9.4 and 72.2°C (40.0±14.79°C). Only the temperature in *Legionella*-positive samples was statistically significantly lower (*P* = 0.012) than in *Legionella*-negative samples (Table 4). In FLA-positive samples temperature (*P* = 0.039) and pH (*P* = 0.041) were statistically significantly lower than in FLA-negative samples. The presence of *Legionella* in compost samples was not statistically significantly correlated with that of FLA (*P* = 0.76; Table 5).

**Table 4:** Physico-chemical characteristic of compost samples positive and negative for *Legionella* or FLA [mean and 95% confidence intervals (95% Cl)].

	Negative		Positive		
	Mean	95% Cl	Mean	95% Cl	*P* value†
***Legionella***					
pH of compost	7.42	7.00–7.85	7.62	7.52–7.71	*P* = 0.23
**Temperature of compost**	53.24	41.83–64.65	38.69	35.26–42.13	***P*** ** = 0.012**
Temperature of air	22.34	16.46–28.22	21.87	20.53–23.20	*P* = 0.84
Relative humidity of air	58.56	41.34–75.77	59.40	55.19–63.61	*P* = 0.91
**FLA**					
**pH of compost**	7.93	7.50–8.35	7.56	7.48–7.67	***P*** ** = 0.041**
**Temperature of compost**	53.18	36.26–70.10	39.10	35.69–42.51	***P*** ** = 0.039**
Temperature of air	20.29	12.09–28.48	22.05	20.78–23.32	*P* = 0.46
Relative humidity of air	59.81	45.47–74.16	59.28	55.02–63.55	*P* = 0.93

† Student t test; *P* values <0.05 were considered statistically significant.

**Table 5:** Frequency of presence of FLA and temperature (<50°C and >50°C) of compost heaps in samples positive or negative for *Legionella*.

		Negative	Positive	Total	*P*-value*
		N (%)	N (%)	N (%)	
**Presence of FLA**					
FLA	Negative	2 (7.4)	5 (8.2)	7 (8)	*P* = 0.76
	Positive	25 (92.6)	56 (91.8)	81 (92)	
**Physical-parameter**					
Temperature	<50°C	4 (57.1)	58 (81.7)	62 (70.4)	*P* = 0.29
	≥50°C	3 (42.9)	13 (18.3)	16 (18.2)	
**Total**				88 (100)	

* Chi-square test with Yates correction; *P* values <0.05 were considered statistically significant.

Air temperatures and relative humidity were not correlated with the presence of *Legionella* in compost samples (Table 4). The air temperatures measured varied between 12.5°C and 33.0°C and the relative humidity between 21.6% to 87.5%.

## Discussion and Conclusions

The results of this study confirmed that the compost is an important reservoir of viable *Legionella* and FLA. The temperature is a promoting factor for *Legionella* and FLA survival in the compost (*P* = 0.012).

*L. pneumophila* and four additional pathogenic species (*L. feeleii*, *L. bozemanii*, *L. longbeachae* and *L. micdadei*) were detected by co-culture in compost samples, with detection limits in the order of 10^2^ *L. pneumophila* in 1 g of compost as described in a previous study [20]. Our data confirm the results of a survey by Casati et al. [25], who, however, never isolated *L. longbeachae*, whereas we detected it in 4.2% of the composts.

Conza et al. have shown that the use of co-culture for bioaerosol analysis allows detecting *Legionella* in samples at concentrations below 10^3^ cells/m^3^ [20]. Using this method, we observed a very low biodiversity in bioaerosols and only *L. pneumophila* sg 1 and *L. pneumophila* other than sg 1 could be isolated.

Twelve and seven amoebal genera (Table 3 and Table S1) were isolated from compost and bioaerosol pools, respectively. FLA supporting intracellular growth of *Legionella* [9] were isolated from both composts and bioaerosols (Table 3). Thus, compost is an important reservoir of FLA, which could be dispersed by the bioaerosols.

We detected *Legionella* and FLA contemporaneously in composts, but we do not know whether or not the *Legionella* isolated were free-living or intracellularly in FLA, thus the isolates from bioaerosol pools were analysed by PCR amplification of the *Legionella mip* gene with the primers (Legmip_f and Legmip_r [26]) to check if *Legionella* are present within amoebae, but all PCR resulted negatives. We did not detect FLA in *Legionella*-positives samples; we did not, however, analyse the bioaerosol samples by 18S or *mip* PCR prior to the isolation of the clones. Although unlikely, we cannot thus exclude that amoebae were present in the *Legionella*-positives samples and were lysed during the overnight storage at 4°C or during the incubation step on NNA agar plate. In our study the presence of *Legionella* in compost was not correlated with the presence of FLA (*P* = 0.76), but these results must be taken with caution because some of the frequencies in the contingency table are too small (Table 5).

The pH of the compost was statistically significantly lower in FLA-positive than FLA-negative samples (*P* = 0.041), as was the compost temperature in samples positive for FLA (*P* = 0.039) and *Legionella* (*P* = 0.012). Nevertheless, *Legionella* was present in 13 composts samples with temperature exceeding 50°C because *Legionella* may survive higher temperature (>50°C) within encysted amoebae [27].

In our investigation, more than two thirds of the compost samples were colonised by *Legionella*: correspondingly, one should expect a high percentage of positive bioaerosols. *Legionella*, however, was isolated only from 10.8% of the bioaerosol pools and we could not detect FLA and *Legionella* simultaneously in the samples. Our results suggest that the pools are fairly representative of the situation in the facilities; nevertheless we are aware that air movements are continuous and microclimatic differences could be present.

A limitation of our results on the presence of *Legionella* in the compost samples is that data derived from a limited number of CF and piles, because the samples were collected from the same pile of compost at each CF at every sample time.

This is the first report of *L. pneumophila* in compost bioaerosols: in previous studies *L. pneumophila* was identified in air samples at a biological treatment plant [28] or from shower baths [29]. *L. pneumophila* was isolated from fewer bioaerosols as compared to FLA: this is probably due to the lower resistance of *Legionella* bacteria to environmental stress factors such as desiccation, UV radiation, and starvation. In addition, non-*pneumophila* species are known to be more susceptible to these factors than *L. pneumophila* [30]. The sampling procedure may also have influenced recovery; the efficacy of *Legionella* and FLA recovery using the Coriolis µ air sampler is not known; in a previous study, the sampling efficacy of this device in a model using *Staphylococcus epidermis* (ATCC 14990) was determined to be 78% [31].

The 2009 sampling season was particularly warm (mean 22.0±5.7°C) and dry compared to previous years and the relative humidity (mean local relative humidity 59.2±18.1%) of the air may have influenced the stability of the bioaerosols and the survival of bacteria and FLA. For example, Berendt reported a lower *L. pneumophila* stability at 50% relative humidity of the air compared to 80% [32]. Furthermore, pathogenic *L. pneumophila* sg 1 strains were least stable at a relative humidity of 60% and survived better than avirulent *L. pneumophila* sg 1 strains [30].

Previous studies reported that bioaerosols containing potential human pathogens may represent a danger for plant workers and nearby residents [17]. Our study has shown that the bioaerosols developed from 3 of the 4 composting facilities analysed contain *L. pneumophila*. Contaminated bioaerosols can be transported by wind [28] and potentially spread to the surrounding areas. In addition, the contaminated compost may be further reused and redistributed in agriculture, gardening or used as potting soil in secondary locations.

In conclusion, our results show that compost could be a reservoir of *Legionella* and FLA. Therefore, composting facilities could be important, possible sources of *Legionella* infections. Further studies are needed to evaluate the extent of the risk to humans deriving from the bioaerosol produced from composting facilities. Investigation of community-acquired LD should include not only water systems and cooling towers but also composting facilities.

## Supporting Information

Table S1 Results of BLAST analysis for 18S rRNA gene sequences of recovered amoebal strains.
(DOCX)

## References

[1] Fliermans CB, Cherry WB, Orrison LH, Smith SJ, Tison DL, et al (1981) Ecological distribution of *Legionella pneumophila* . Appl Environ Microbiol 41: 9–16. 7013702 10.1128/aem.41.1.9-16.1981 PMC243633

[2] Joseph CA, Ricketts KD, on behalf of the European Working Group for *Legionella* Infections (2010) Legionnaires’ disease in Europe 2007–2008. Euro Surveill. Available: http://www.eurosurveillance.org/ViewArticle.aspx?ArticleId=19493. Accessed 23 December 2012. 10.2807/esm.09.10.00480-en 29183473

[3] Steele TW, Moore CV, Sangster N (1990) Distribution of *Legionella longbeachae* serogroup 1 and other legionellae in potting soils in Australia. Appl Environ Microbiol 56: 2984–2988. 2285311 10.1128/aem.56.10.2984-2988.1990 PMC184887

[4] den Boer JW, Yzerman EP, Jansen R, Bruin JP, Verhoef LP, et al (2007) Legionnaires’ disease and gardening. Clin Microbiol Infect 13: 88–91. 17184293 10.1111/j.1469-0691.2006.01562.x

[5] Casati S, Gioria-Martinoni A, Gaia V (2009) Commercial potting soils as an alternative infection source of *Legionella pneumophila* and other *Legionella* species in Switzerland. Clin Microbiol Infect 15: 571–575. 19392903 10.1111/j.1469-0691.2009.02742.x

[6] Nguyen TM, Ilef D, Jarraud S, Rouil L, Campese C, et al (2006) A community-wide outbreak of legionnaires disease linked to industrial cooling towers–how far can contaminated aerosols spread? J Infect Dis 193: 102–111. 16323138 10.1086/498575

[7] Lin H, Xu B, Chen Y, Wang W (2009) *Legionella* pollution in cooling tower water of air-conditioning systems in Shanghai, China. J Appl Microbiol 106: 606–612. 19120608 10.1111/j.1365-2672.2008.04031.x

[8] Greub G, Raoult D (2004) Microorganisms resistant to free-living amoebae. Clin Microbiol Rev 17: 413–433. 15084508 10.1128/CMR.17.2.413-433.2004 PMC387402

[9] Dey R, Bodennec J, Mameri MO, Pernin P (2009) Free-living freshwater amoebae differ in their susceptibility to the pathogenic bacterium *Legionella pneumophila* . FEMS Microbiol Lett 290: 10–17. 19016880 10.1111/j.1574-6968.2008.01387.x

[10] Schuster FL (2002) Cultivation of pathogenic and opportunistic free-living amebas. Clin Microbiol Rev 15: 342–354. 12097243 10.1128/CMR.15.3.342-354.2002 PMC118083

[11] Rowbotham TJ (1983) Isolation of *Legionella pneumophila* from clinical specimens via amoebae, and the interaction of those and other isolates with amoebae. J Clin Pathol 36: 978–986. 6350372 10.1136/jcp.36.9.978 PMC498455

[12] Thomas V, Herrera-Rimann K, Blanc DS, Greub G (2006) Biodiversity of amoebae and amoeba-resisting bacteria in a hospital water network. Appl Environ Microbiol 72: 2428–2438. 16597941 10.1128/AEM.72.4.2428-2438.2006 PMC1449017

[13] Descours G, Suet A, Ginevra C, Campese C, Slimani S, et al (2012) Contribution of Amoebic Coculture to Recovery of *Legionella* Isolates from Respiratory Samples: Prospective Analysis over a Period of 32 Months. J Clin Microbiol 50: 1725–1726. 22322354 10.1128/JCM.06531-11 PMC3347135

[14] Declerck P, Behets J, van Hoef V, Ollevier F (2007) Detection of *Legionella* spp. and some of their amoeba hosts in floating biofilms from anthropogenic and natural aquatic environments. Water Res 41: 3159–3167. 17544473 10.1016/j.watres.2007.04.011

[15] Danon M, Franke-Whittle IH, Insam H, Chen Y, Hadar Y (2008) Molecular analysis of bacterial community succession during prolonged compost curing. FEMS Microbiol Ecol 65: 133–144. 18537836 10.1111/j.1574-6941.2008.00506.x

[16] Sanchez-Monedero MA, Stentiford EI, Urpilainen ST (2005) Bioaerosol generation at large-scale green waste composting plants. J Air Waste Manag Assoc 55: 612–618. 15991670 10.1080/10473289.2005.10464652

[17] Fracchia L, Pietronave S, Rinaldi M, Martinotti MG (2006) The assessment of airborne bacterial contamination in three composting plants revealed site-related biological hazard and seasonal variations. J Appl Microbiol 100: 973–984. 16629998 10.1111/j.1365-2672.2006.02846.x

[18] Taha MP, Pollard SJ, Sarkar U, Longhurst P (2005) Estimating fugitive bioaerosol releases from static compost windrows: feasibility of a portable wind tunnel approach. Waste Manag 25: 445–450. 15869988 10.1016/j.wasman.2005.02.013

[19] Hryhorczuk D, Curtis L, Scheff P, Chung J, Rizzo M, et al (2001) Bioaerosol emissions from a suburban yard waste composting facility. Ann Agric Environ Med 8: 177–185. 11748875

[20] Conza L, Casati S, Gaia V (2013) Detection limits of *Legionella pneumophila* in environmental samples after co-culture with *Acanthamoeba polyphaga* . BMC Microbiol 13: 49. 23442526 10.1186/1471-2180-13-49 PMC3598970

[21] La Scola B, Mezi L, Weiller PJ, Raoult D (2001) Isolation of *Legionella anisa* using an amoebic coculture procedure. J Clin Microbiol 39: 365–366. 11136802 10.1128/JCM.39.1.365-366.2001 PMC87733

[22] Gaia V, Casati S, Tonolla M (2011) Rapid identification of *Legionella* spp. by MALDI-TOF MS based protein mass fingerprinting. Syst Appl Microbiol 34: 40–44. 21247716 10.1016/j.syapm.2010.11.007

[23] Helbig JH, Bernander S, Castellani Pastoris M, Etienne J, Gaia V, et al (2002) Pan-European study on culture-proven Legionnaires’ disease: distribution of *Legionella pneumophila* serogroups and monoclonal subgroups. Eur J Clin Microbiol Infect Dis 21: 710–716. 12415469 10.1007/s10096-002-0820-3

[24] Helbig JH, Kurtz JB, Pastoris MC, Pelaz C, Luck PC (1997) Antigenic lipopolysaccharide components of *Legionella pneumophila* recognized by monoclonal antibodies: possibilities and limitations for division of the species into serogroups. J Clin Microbiol 35: 2841–2845. 9350744 10.1128/jcm.35.11.2841-2845.1997 PMC230072

[25] Casati S, Conza L, Bruin J, Gaia V (2009) Compost facilities as a reservoir of *Legionella pneumophila* and other *Legionella* species. Clin Microbiol Infect 16: 945–947. 19645762 10.1111/j.1469-0691.2009.03009.x

[26] Ratcliff RM, Lanser JA, Manning PA, Heuzenroeder MW (1998) Sequence-based classification scheme for the genus *Legionella* targeting the *mip* gene. J Clin Microbiol 36: 1560–1567. 9620377 10.1128/jcm.36.6.1560-1567.1998 PMC104877

[27] Storey MV, Winiecka-Krusnell J, Ashbolt NJ, Stenstrom TA (2004) The efficacy of heat and chlorine treatment against thermotolerant *Acanthamoebae* and *Legionellae* . Scand J Infect Dis 36: 656–662. 15370652 10.1080/00365540410020785

[28] Blatny JM, Reif BA, Skogan G, Andreassen O, Hoiby EA, et al (2008) Tracking airborne *Legionella* and *Legionella pneumophila* at a biological treatment plant. Environ Sci Technol 42: 7360–7367. 18939571 10.1021/es800306m

[29] Dennis PJ, Wright AE, Rutter DA, Death JE, Jones BP (1984) *Legionella pneumophila* in aerosols from shower baths. J Hyg (Lond) 93: 349–353. 6501881 10.1017/s0022172400064901 PMC2129433

[30] Dennis PJ, Lee JV (1988) Differences in aerosol survival between pathogenic and non-pathogenic strains of *Legionella pneumophila* serogroup 1. J Appl Bacteriol 65: 135–141. 3204070 10.1111/j.1365-2672.1988.tb01501.x

[31] Carvalho E, Sindt C, Verdier A, Galan C, O’Donoghue L, et al (2008) Performance of the Coriolis air sampler, a high-volume aerosol-collection system for quantification of airborne spores and pollen grains. Aerobiologia 24: 191–201.

[32] Berendt RF (1980) Survival of *Legionella pneumophila* in aerosols: effect of relative humidity. J Infect Dis 141: 689. 7373091 10.1093/infdis/141.5.689

[33] Rodriguez-Zaragoza S (1994) Ecology of free-living amoebae. Crit Rev Microbiol 20: 225–241. 7802958 10.3109/10408419409114556

