# Supplementary material for: Presence of Legionella and Free-Living Amoebae in Composts and Bioaerosols from Composting Facilities
Source: PLoS One. 2013 Jul 2;8(7):e68244. doi: 10.1371/journal.pone.0068244 (PMC3699541; doi:10.1371/journal.pone.0068244)
Supplement: Table S1 — Results of BLAST analysis for 18S rRNA gene sequences of recovered amoebal strains. (DOCX) [file pone.0068244.s001.docx]

| **Closest previously described species** | **Strain isolated in this study** | | | |
| --- | --- | --- | --- | --- |
| **Specie (GenBank Accession No.)** | **Strain (GenBank Accession No.)^a^** | **Origin** | **Nucleotide identity (%)^b^** | **Isolates** |
| *Acanthamoeba castellanii* (AF114438) | CF3-107 (KC164230) | compost | 99% | 3 |
| *Acanthamoeba castellanii* 4CL (AF260724) | CF1-119b (KC164234) | compost | 99% | 1 |
| *Acanthamoeba hatchetti* BH-2 (AF019068) | CF2-46 (KC164222) | compost | 99% | 2 |
| *Acanthamoeba hatchetti* 4RE (AF251937) | CF1-120 (KC164235) | compost | 99% | 2 |
| *Acanthamoeba lenticulata* (U94734) | CF1-249 (KC164253) | compost | 100% | 2 |
| *Acanthamoeba polyphaga* JAC (U07415) | CF3-77b (KC164226) | compost | 99% | 2 |
| *Acanthamoeba* sp. EFW4 (DQ992185) | CF3-58 (KC164223) | compost | 99% | 1 |
| *Acanthamoeba* sp. AcaVNAK03 (GQ905497) | CF3-78 (KC164227) | compost | 99% | 1 |
| *Acanthamoeba* sp. KA/E19 (EF140631) | CF4-114 (KC164232) | compost | 99% | 1 |
| *Acanthamoeba* sp. KA/E7 (AY148955) | CF3-125b (KC164237) | compost | 100% | 2 |
| *Acanthamoeba* sp. environmental clone (EF023783) | CF4-66 (KC210135) | compost | 99% | 1 |
| *Acanthamoeba* sp. Liu-E1 (AF019055) | CF4-114 (KC210137) | compost | 99% | 1 |
| *Acanthamoeba* sp. AcaVN08 (GQ397470) | CF4-132 (KC210134) | compost | 99% | 2 |
| *Acanthamoeba* sp. MZOR (DQ103890) | CF2-252 (KC210136) | aerosol | 99% | 1 |
| *Echinamoeba thermatum* (AJ489267)* | CF4-238 (KC164252) | compost | 75% | 1 |
| *Learamoeba waccamawenis* (AF011455) | CF1-138 (KC164239) | compost | 99% | 2 |
| *Hartmannella vermiformis* (AB525836) | CF4-113 (KC164231) | compost | 100% | 2 |
| *Hartmannella vermiformis* KWR-1 (AY502960) | CF3-181 (KC164244) | compost | 99% | 1 |
| *Hartmannella vermiformis* CRIB06 (DQ123623) | CF4-197 (KC164245) | compost | 99% | 3 |
| *Hartmannella vermiformis* clone 4391 (DQ084364) | CF3-226 (KC164250) | compost | 100% | 2 |
| *Hartmannella vermiformis* (AB525839) | CF4-83 (KC210138) | compost | 99% | 1 |
| *Hartmannella vermiformis* S077-R3 (FJ628003) | CF1-246 (KC210139) | aerosol | 93% | 2 |
| *Hartmanella* sp. 2 4/3DA/10 (AY680841) | CF3-166 (KC164243) | compost | 96% | 1 |
| *Naegleria australiensis* (U80058) | CF4-34 (KC164221) | compost | 100% | 2 |
| *Naegleria australiensis* (U80058) | CF3-125 (KC164236) | compost | 100% | 5 |
| *Naegleria* cf. *gruberi* isolate 6i (FJ948856) | CF3-126 (KC164238) | compost | 99% | 1 |
| *Naegleria gruberi* (AB298288) | CF3-203 (KC164247) | compost | 100% | 3 |
| *Naegleria gruberi* sp. ATCC 30294 (AF011457) | CF3-126 (KC210140) | compost | 99% | 2 |
| *Naegleria* sp. CRIB62 (EU377593) | CF3-15 (KC164220) | compost | 100% | 6 |
| *Naegleria* sp. NG260 (AM071385) | CF4-84 (KC164228) | compost | 99% | 1 |
| *Naegleria* sp. NG 152 (AM062739) | CF3-40 (KC210141) | compost | 100% | 1 |
| *Singhamoeba horticola* (AF011456) | CF2-102 (KC164229) | compost | 97% | 1 |
| Soil amoeba AND32 (AY96587) | CF4-66 (KC164224) | compost | 99% | 1 |
| *Vahlkampfia avara* (AJ224886) | CF1-119 (KC164233) | compost | 99% | 1 |
| *Vahlkampfia avara* (AJ224886) | CF1-138b (KC164240) | compost | 99% | 1 |
| *Vahlkampfia avara* (AJ224886) | CF1-144 (KC164241) | compost | 99% | 1 |
| *Vahlkampfia avara* (AJ224886) | CF3-154 (KC164242) | compost | 99% | 2 |
| *Vahlkampfia enterica* (AJ224889) | CF1-11 (KC164219) | compost | 97% | 4 |
| *Willaertia magna* (AY266315) | CF3-77a (KC164225) | compost | 100% | 1 |
| *Willaertia magna* (AY266315) | CF3-202 (KC164246) | compost | 100% | 1 |
| *Acanthamoeba jacobsi* AC305 (AY262365) | CF1-219 (KC164249) | aerosol | 100% | 3 |
| *Flamella balnearia* (EU186023) | CF1-210 (KC164248) | aerosol | 94% | 3 |
| *Naegleria americana* (AM062740) | CF2-1 (KC164218) | aerosol | 100% | 1 |
| *Platyamoeba placida* (AY294150) | CF4-234 (KC164251) | aerosol | 94% | 1 |
| *Stenamoeba* sp. (EU377587) | CF3-16 (KC210143) | compost | 99% | 25 |
| *Stenamoeba* sp. (EU377587) | CF4-196 (KC210145) | compost | 98% | 25 |
| *Tetramitus* sp. F8-15 (EF378688) | CF3-107 (KC210142) | compost | 100% | 2 |

1. The accession numbers are GenBank accession numbers. BLAST analysis was used to determine the level of 18S rRNA gene sequence homology with the most similar GenBank sequence.
2. 18S rRNA gene homology with closest GenBank described species.

**Echinamoeba thermatum* has a nucleotide identity of 75% with a corresponding database entry, which presumably is even not sufficient for a reliable identification at the genus level.
